# Long-Term Continuous Cervical Spinal Cord Stimulation Exerts Neuroprotective Effects in Experimental Parkinson’s Disease

**DOI:** 10.3389/fnagi.2020.00164

**Published:** 2020-06-16

**Authors:** Ken Kuwahara, Tatsuya Sasaki, Takao Yasuhara, Masahiro Kameda, Yosuke Okazaki, Kakeru Hosomoto, Ittetsu Kin, Mihoko Okazaki, Satoru Yabuno, Satoshi Kawauchi, Yousuke Tomita, Michiari Umakoshi, Kyohei Kin, Jun Morimoto, Jea-Young Lee, Naoki Tajiri, Cesar V. Borlongan, Isao Date

**Affiliations:** ^1^Department of Neurological Surgery, Graduate School of Medicine, Dentistry and Pharmaceutical Sciences, Okayama University, Okayama, Japan; ^2^Department of Neurosurgery and Brain Repair, Morsani College of Medicine, University of South Florida, Tampa, FL, United States; ^3^Department of Neurophysiology and Brain Science, Graduate School of Medical Sciences, Nagoya City University, Nagoya, Japan

**Keywords:** electrical stimulation, neuroinflammation, neuromodulation, neuroprotection, 6-hydroxydopamine

## Abstract

**Background:**

Spinal cord stimulation (SCS) exerts neuroprotective effects in animal models of Parkinson’s disease (PD). Conventional stimulation techniques entail limited stimulation time and restricted movement of animals, warranting the need for optimizing the SCS regimen to address the progressive nature of the disease and to improve its clinical translation to PD patients.

**Objective:**

Recognizing the limitations of conventional stimulation, we now investigated the effects of continuous SCS in freely moving parkinsonian rats.

**Methods:**

We developed a small device that could deliver continuous SCS. At the start of the experiment, thirty female Sprague-Dawley rats received the dopamine (DA)-depleting neurotoxin, 6-hydroxydopamine, into the right striatum. The SCS device was fixed below the shoulder area of the back of the animal, and a line from this device was passed under the skin to an electrode that was then implanted epidurally over the dorsal column. The rats were divided into three groups: control, 8-h stimulation, and 24-h stimulation, and behaviorally tested then euthanized for immunohistochemical analysis.

**Results:**

The 8- and 24-h stimulation groups displayed significant behavioral improvement compared to the control group. Both SCS-stimulated groups exhibited significantly preserved tyrosine hydroxylase (TH)-positive fibers and neurons in the striatum and substantia nigra pars compacta (SNc), respectively, compared to the control group. Notably, the 24-h stimulation group showed significantly pronounced preservation of the striatal TH-positive fibers compared to the 8-h stimulation group. Moreover, the 24-h group demonstrated significantly reduced number of microglia in the striatum and SNc and increased laminin-positive area of the cerebral cortex compared to the control group.

**Conclusions:**

This study demonstrated the behavioral and histological benefits of continuous SCS in a time-dependent manner in freely moving PD animals, possibly mediated by anti-inflammatory and angiogenic mechanisms.

## Introduction

Parkinson’s disease manifests as a progressive neurodegenerative disease resulting from the loss of dopaminergic neurons in the nigrostriatal system. Cardinal symptoms of PD include bradykinesia, rigidity, resting tremor, and postural instability. Levodopa treatment stands as the first-line therapy for PD. However, long-term medication often results in adverse events, including motor fluctuation and dyskinesia.

Deep brain stimulation (DBS) improves motor symptoms in advanced PD patients. In animal models of PD, DBS may increase BDNF (Spieles-Engemann et al., 2010) and may prevent DA neuron loss in the SNc (Maesawa et al., 2004; Spieles-Engemann et al., 2011). However, DBS entails an invasive surgical procedure that damages brain tissue and involves a permanent system implant. The estimated risk of intracranial hemorrhage in DBS ranges from 0.8 to 2.8% (Obeso et al., 2001; Herzog et al., 2003; Sansur et al., 2007; Weaver et al., 2009; Fenoy and Simpson Jr., 2014). Moreover, the efficacy of DBS appears effective only in cases with motor fluctuation responsive to levodopa therapy, thus, limited PD patients are eligible for DBS.

Spinal cord stimulation in the management of intractable neuropathic pain demonstrates a solid track record of effectiveness and safety. Although neurological injuries account for the most serious complication in SCS procedure, they are rare with an incidence rate of only 0.6% (Levy et al., 2011). In animal models of PD, SCS alleviates motor deficits (Fuentes et al., 2009; Santana et al., 2014; Shinko et al., 2014; Yadav et al., 2014) and protects nigrostriatal dopaminergic neurons (Fuentes et al., 2009; Shinko et al., 2014). In advanced PD patients with lumbago and leg pain, SCS improves motor function such as posture, postural stability, and gait ability (Agari and Date, 2012).

Electrical stimulation shows efficacy in PD animal models. However, technical problems plague the SCS animal model, including the short duration of the stimulation (no more than 1 h per day) and the highly restricted movement of animals (i.e., due to anesthesia) (Maesawa et al., 2004; Boulet et al., 2006; Spieles-Engemann et al., 2010, 2011; Shinko et al., 2014; Yadav et al., 2014; Huotarinen et al., 2018). The advent of small mobile stimulators enables continuous DBS in freely moving parkinsonian rats (Badstübner et al., 2012; Badstuebner et al., 2017). Cognizant of SCS in PD animal models not closely replicating the clinical application, customizing the small mobile stimulators used in DBS for SCS may overcome these preclinical limitations. To date, continuous SCS in freely moving PD animals remains unexplored. In the present study, we developed a small mobile device for continuous SCS in freely moving parkinsonian rats.

## Materials and Methods

### Animals and Animal Care

All animal procedures in this study followed specifically the approved guidelines by the Institutional Animal Care and Use Committee of Okayama University Graduate School of Medicine (Protocol# OKU-2018807). Adult female Sprague-Dawley rats (Shimizu Laboratory Supplies Co., Ltd., Japan) weighing 200–250 g at the beginning of the study served as subjects for all experiments. Animal housing consisted of individual cages in a temperature and humidity-controlled room and maintained on a semidiurnal light-dark cycle.

### Small Mobile Device for Continuous Electrical Stimulation

We developed an electrical stimulation device called SAS-200 (Unique Medical Co., Ltd., Japan) that offered convenient adjustment of stimulation conditions via Bluetooth and allowed free movement of rats owing to its small size. The SAS-200SCS, which was attached to the back of the rats and connected to the SCS electrode, delivered the stimulation. This stimulation required no anesthesia, thereby allowing rats to freely move around, making continuous stimulation possible. Additionally, the stimulation conditions could be easily adjusted wirelessly.

**FIGURE 1:**
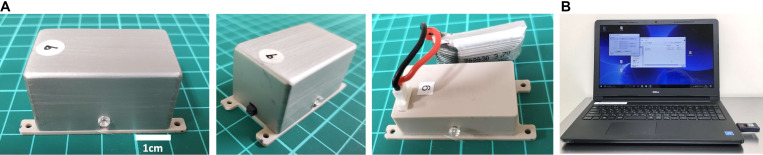
Wireless controllable electrical stimulation system (SAS-200). **(A)** The stimulation device measures 20 mm × 40 mm × 20 mm, with a net weight of 26 g (including the battery). The control panel is covered by an aluminum case and fixed by screws on two sides. **(B)** Stimulation conditions can be changed using a Windows PC and transmitted through Bluetooth.

The SAS-200 measured 20 mm × 40 mm × 20 mm, with a net weight of 26 g (including the battery) (Figure 1A). It consisted of a control panel, a rechargeable lithium-ion battery, and an aluminum case. An aluminum case covered the unit and fixed by screws on two sides. The SAS-200 generated biphasic square pulses with stimulation conditions programmed in the control panel, and as many as 1,650 patterns of stimulation could be adjusted accordingly. Based on pilot stimulation optimization studies, we selected 10 stimulation parameters for stimulation intensity (0.25, 0.4, 0.5, 0.6, 0.7, 0.8, 1.0, 1.2, 1.5, and 2.0 mA), 11 for frequency (1, 2, 5, 10, 20, 30, 50, 100, 150, 200, and 300 Hz), three for pulse width (100, 250, and 500 μs), and five for stimulation cycle [(A) continuous stimulation, (B) 8 h on 16 h off, (C) 12 h on 12 h off, (D) 30 s on 5 min off, and (E) 15 trains every 12 s]. A standard Windows PC with a specific application controlled these stimulation conditions, such as beginning, duration, and particular conditions (Figure 1B). An LED light, which was placed below the transparent screw on the right side, served as the stimulation and battery indicator; when Bluetooth initiated the stimulation, the light turned on, and the light flickered when the battery dropped below 20%. We used a rechargeable battery with an AC adaptor. In our experiments, we fixed and encased in a protective jacket the SAS-200 to the back of the animals through threads at four fixing holes. A battery change involved simply removing the screws and replacing the depleted battery with a fully charged battery.

### Experimental Design

**FIGURE 2:**
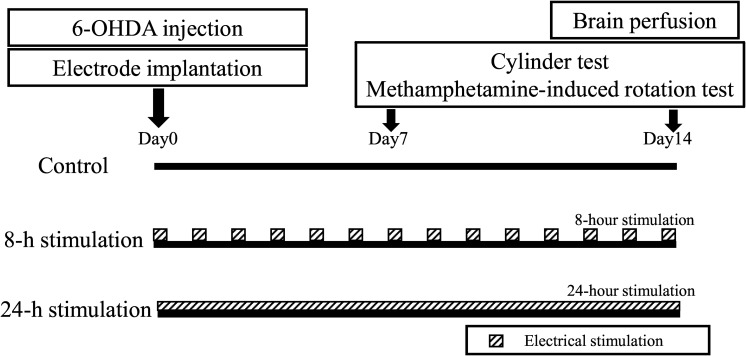
Time course of this study.

Rats were randomly divided into three groups: the control, 8-h stimulation, and 24-h stimulation groups (30 rats total, *n* = 10 in each group) (see study time course in Figure 2). On day 0, all rats received 6-OHDA, which was injected into the right striatum. Subsequently, all rats underwent C2 laminectomy and implanted with an electrode in their epidural space, with the external mobile stimulator subsequently attached to their back. After recovery from anesthesia, stimulation commenced in the 8- and 24-h stimulation groups (see detailed stimulation protocol below). On days 7 and 14, all rats received behavioral tests, and thereafter euthanized for immunohistochemical investigations and morphological analyses.

### Surgical Procedure

#### 6-OHDA Lesioning

All rats received anesthesia with 0.3 mg/kg of medetomidine, 4.0 mg/kg of midazolam, and 5.0 mg/kg of butorphanol by intraperitoneal injection and placed in a stereotaxic instrument (Narishige, Japan). The animals underwent a midline head skin incision on and a small hole drilled in their skull. Twenty μg of 6-OHDA (4 μl of 5 mg/ml dissolved in saline containing 0.2 mg/ml of ascorbic acid; Sigma, United States) was injected into the right striatum (1.0 mm anterior and 3 mm lateral to the bregma and 5.0 mm ventral to the surface of the brain with the tooth-bar set at −1.0 mm) with a 28G Hamilton syringe that delivered an injection rate of the drug at 1 μl/min. Syringe withdrawal commenced after a 5-min absorption time following injection.

#### Implantation of Stimulation Electrode

**FIGURE 3:**
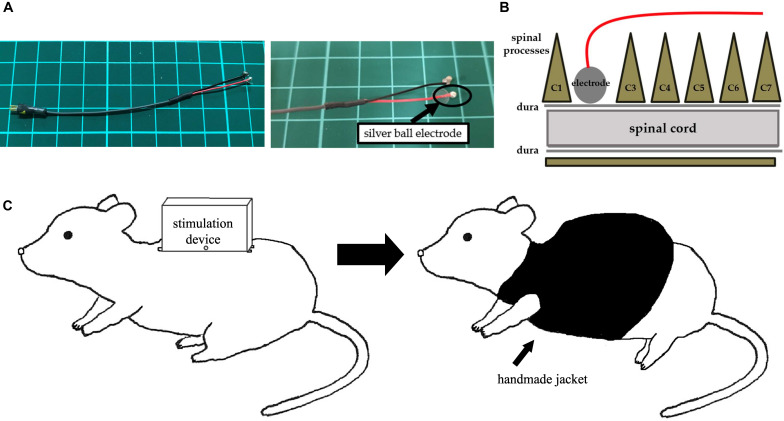
An electrode and images of surgery. **(A)** A silver ball SCS electrode used in this study (diameter: 2 mm). **(B)** An image showing electrode implantation. A silver ball electrode was placed on the dorsal surface of the spinal cord and fixed by a silk thread. **(C)** An image showing a rat with a stimulation device. After fixation of the stimulation device on the back, a handmade jacket was put on the rat.

Following 6-OHDA injection, animals received a midline skin incision that extended to the back, and carefully dissecting the spinal muscles to expose and to eventually perform a C2 laminectomy. We implanted a silver bipolar ball electrode, with a diameter of 2 mm, epidurally on the dorsal surface of the spinal cord and fixed to the muscle using a 5-0 silk thread (Figures 3A,B). We then placed a ground electrode in the skull of the rat, with the lead tunneled subcutaneously to the back of rats. Finally, the rats received the stimulation device that was fixed on their back using 1-0 silk threads at four fixing holes and encased in a protective jacket (Figure 3C).

### Electrical Stimulation

After recovery from anesthesia, the stimulation device commenced by wireless command from Windows PC via Bluetooth in the 8- and 24-h stimulation groups. In the 8-h stimulation group, the stimulator automatically delivered biphasic square pulses for 8 h then switched off for 16 h. Stimulation continued for 14 consecutive days, and with battery changed every 3 days. Stimulation consisted of 50 Hz pulses in 100 μs. Intensities corresponded to the 80% of motor threshold (Supplementary Video S1). The parameter was determined based on the results of our previous studies demonstrating neuroprotective effects for PD model rats (Shinko et al., 2014).

### Behavioral Tests

#### Cylinder Test

To assess the degree of forepaw asymmetry, we performed the cylinder test on days 7 and 14. This test involved placing individual animals in a transparent cylinder (diameter: 20 cm, height: 30 cm) for 3 min and recording the number of forepaw contacts on the cylinder wall (Schallert et al., 2000; Shinko et al., 2014). The score of the cylinder test reflected a contralateral bias: ([number of contacts with contralateral limb] − [number of contacts with ipsilateral limb]/[number of total contacts] × 100) (Roof et al., 2001; Shinko et al., 2014; Sasaki et al., 2016). This contralateral bias indicated successful 6-OHDA-induced unilateral depletion of nigrostriatal dopaminergic neurons and fibers.

#### Methamphetamine-Induced Rotation Test

Rats received an intraperitoneal injection of methamphetamine (3.0 mg/kg; Dainippon Sumitomo Pharma, Japan) on days 7 and 14. We assessed for 90 min with a video camera the full 360° turns in the direction ipsilateral to the lesion. Such drug-induced ipsilateral rotations also indicated successful 6-OHDA-induced unilateral nigrostriatal dopaminergic depletion.

### Immunohistochemical Investigations

Processing for immunohistochemistry started after completion of behavioral tests on day 14. Animals underwent euthanasia with an overdose of pentobarbital (100 mg/kg). The rats then received transcardial perfusion with 150 ml of cold phosphate-buffered saline (PBS) and 150 ml of 4% paraformaldehyde (PFA) in PBS. We then harvested the brains carefully, post fixed in 4% PFA in PBS overnight at 4°C, and subsequently stored in 30% sucrose in PBS until completely submerged. Thereafter, we sectioned the brains coronally at a thickness of 40 μm.

For assessing nigrostriatal dopaminergic pathways, we used TH staining. We initially exposed free-floating sections to a blocking solution using 3% hydrogen peroxide in 70% methanol for 7 min. After three washes in PBS, we incubated the sections overnight at 4°C, with rabbit anti-TH antibody (1:500; Chemicon, Temecula, CA, United States) with 10% normal horse serum. We then washed the sections three times for 5 min in PBS and incubated them for 1 h in biotinylated donkey anti-rabbit IgG (1:500; Jackson ImmunoResearch Lab, West Grove, PA, United States), followed by 30 min in avidin-biotin-peroxidase complex (Vector Laboratories, Burlingame, CA, United States). We next treated the sections with 3, 4-diaminobenzidine (DAB; Vector) and hydrogen peroxide, then mounted on albumin-coated slides, and embedded them with cover glass.

Next, we performed Iba-1 and laminin staining to evaluate activated microglial cells and blood vessels, respectively. We initially washed 40-μm-thick sections three times in PBS and incubating them in 10% normal horse serum and primary antibodies: rabbit anti-Iba1 antibody (1:250; Wako Pure Chemical Industries, Osaka, Japan) and rabbit anti-laminin antibody (1:500; AB11575, Abcam plc, Cambridge, United Kingdom) overnight at 4°C. Thereafter, we washed the sections three times in PBS, incubated them for 1 h in FITC-conjugated affinity-purified donkey anti-rabbit IgG (H + L) in a dark chamber, then washed them three more times in PBS and finally mounted and embedded them with cover glass as above.

### Morphological Analyses

We assessed the density of TH-positive fibers in the striatum with a computerized analysis system as described previously (Shinko et al., 2014). Investigators blinded to the treatment conditions randomly selected three sections at 0.5, 1.0, and 1.5 mm anterior to the bregma for quantitative analysis. The two areas adjacent to the needle tract of the lesion side and the symmetrical areas in the contralateral side served as the brain region of interest. We then converted the brain photographs into binary images using an appropriate threshold (Image J; National Institutes of Health, Bethesda, MD, United States), and calculated the percentages of the lesion to the intact side in each section, with the averages subsequently used for statistical analyses. We counted all the number of TH-positive dopaminergic neurons in three sections at 4.8, 5.3, and 5.8 mm posterior to the bregma in the SNc, but not in the ventral tegmental area. We then calculated the percentage of the lesioned side to the intact side, then using the averages for the statistical analyses. We also counted the number of Iba-1 positive cells with nuclei in the lesion side of the striatum and SNc using randomly selected fixed areas (500 μm × 500 μm square) from two different sections (0.5 and 1.0 mm anterior to the bregma), then used the averages used for statistical analyses. Additionally, we measured the area of laminin-positive structures as percentages relative to the area of the randomly captured images (500 μm × 500 μm square) from two different sections of the cortex (4 mm lateral to the midline and 0.5 and 1.0 mm anterior to the bregma) then also used the averages for statistical analyses.

### Statistical Analyses

We used the software package SPSS 20.0 (SPSS, Chicago, IL, United States) to perform one-way analysis of variance (ANOVA) with subsequent Tukey’s tests, with significance set at *p* < 0.05. Data showed here represented means ± standard deviation (SD).

## Results

### Body Weight

**FIGURE 4:**
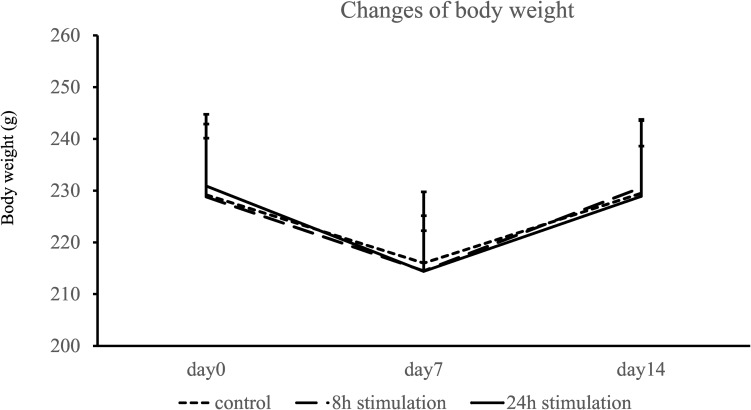
Changes in body weight.

Body weight decreased at day 7 and nearly recovered at day 14 in all groups (Figure 4). Body weights did not significantly differ on days 0, 7, and 14 between the control, 8-, and 24-h stimulation groups (body weight on days 0, 7, and 14: control group: 229.2 ± 13.7, 216.0 ± 13.8, and 229.5 ± 14.0 g; 8-h stimulation group: 228.8 ± 11.3, 214.5 ± 7.76, and 230.6 ± 8.01 g; and 24-h stimulation group: 230.9 ± 13.8, 214.4 ± 10.8, and 228.9 ± 14.9 g, respectively).

### Behavioral Tests

#### Cylinder Test

**FIGURE 5:**
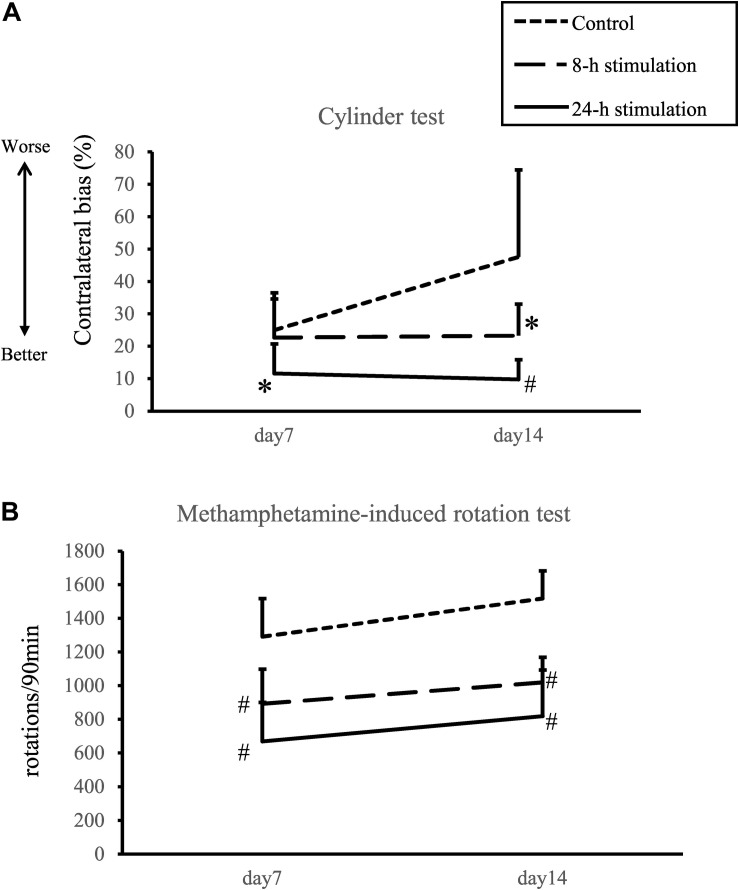
Spinal cord stimulation and behavioral outcomes. **(A)** Contralateral bias in the cylinder test. In the 24-h stimulation group, improvement of contralateral bias was observed from days 7 to 14. In the 8-h stimulation group, improvement was observed on day 14 (^#^*p* < 0.01, ^∗^*p* < 0.05). **(B)** Methamphetamine-induced rotations per 90 min. The number of methamphetamine-induced rotations significantly decreased in the 8- and 24-h stimulation groups compared to the control group (^#^*p* < 0.01).

The 24-h stimulation group performed significantly better in the cylinder test than the control group on days 7 and 14. In the 8-h stimulation group, the treated animals displayed significant improvement in the contralateral bias on day 14 compared to the control group (contralateral bias: control group: 25.0 ± 10.1 and 47.6 ± 28.4%; 8-h stimulation group: 22.7 ± 14.7 and 23.3 ± 12.3%; 24-h stimulation group: 11.6 ± 9.56 and 9.80 ± 6.39% at 1 and 2 weeks, respectively; Figure 5A). For comparison, the contralateral bias before 6-OHDA lesion was 1.3 ± 0.8%.

#### Methamphetamine-Induced Rotation Test

The number of methamphetamine-induced rotations on days 7 and 14 in the 8- and 24-h stimulation groups statistically decreased compared to that of the control group (control group: 1292 ± 239 and 1518 ± 172 turns/90 min; 8-h stimulation group: 893 ± 217 and 1,020 ± 146 turns/90 min; 24-h stimulation group: 670 ± 244 and 820 ± 289 turns/90 min at 1 and 2 weeks, respectively; Figure 5B). The 8- and 24-h stimulation groups did not significantly differ in their rotational behaviors. For comparison, the rotational number before 6-OHDA lesion was 18 ± 10 turns/90 min.

### Immunohistochemical Investigations

#### TH (Tyrosine Hydroxylase) Staining

**FIGURE 6:**
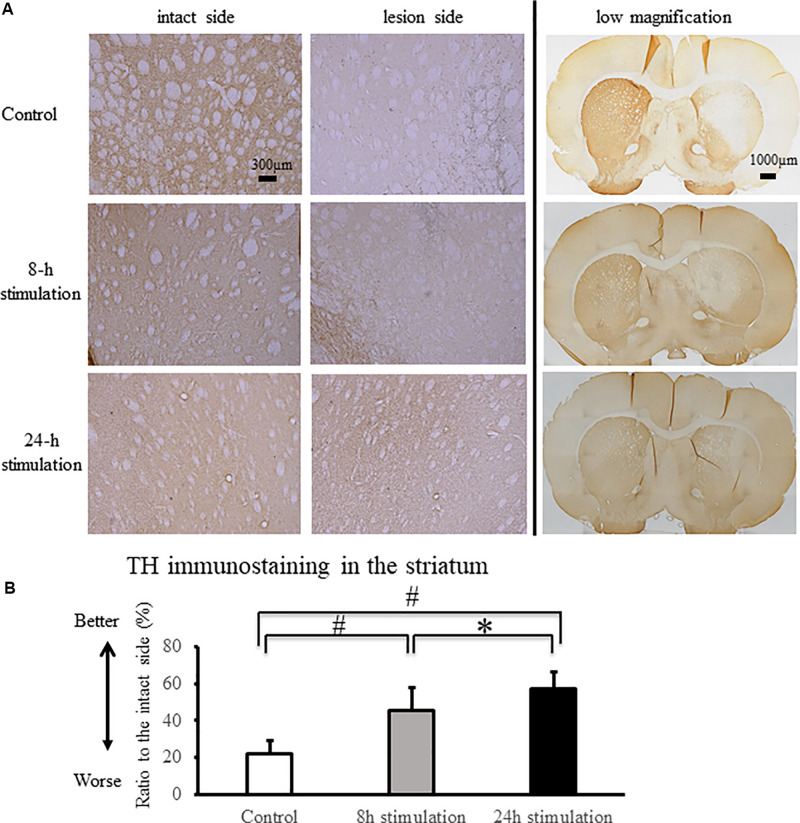
Spinal cord stimulation and TH staining in the striatum. **(A)** TH-positive fibers were preserved in the striatum of the 8- and 24-h stimulation groups (10×). In the right column, the low magnified images are shown (2×). **(B)** The ratio of TH-positive fibers in the lesioned striatum to the intact side was significantly preserved in the stimulation groups compared to that in the control group (^#^*p* < 0.01). TH-positive fibers in the striatum of rats in the 24-h stimulation group were significantly preserved compared to those in the 8-h stimulation group (^∗^*p* < 0.05).

**FIGURE 7:**
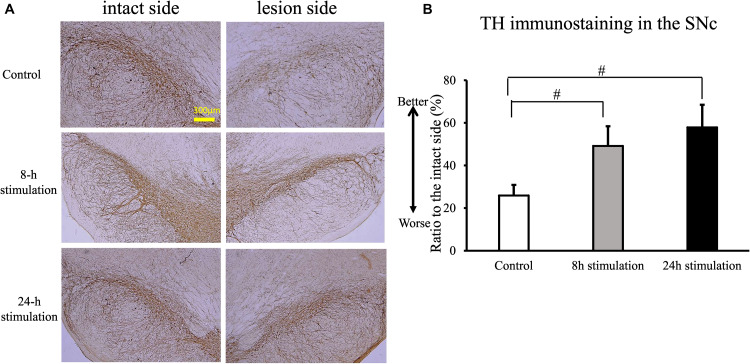
Spinal cord stimulation and TH staining in the SNc. **(A)** TH-positive neurons in the SNc were preserved in the stimulation groups (10×). **(B)** TH-positive neurons in the SNc in the 8- and 24-h stimulation groups were significantly preserved compared to those in the control group (^#^*p* < 0.01).

The stimulation groups exhibited significant preservation of TH-positive fibers in the striatum and TH-positive neurons in the SNc compared to the control group (control group: 21.9 ± 7.16%; 8-h stimulation group: 45.3 ± 12.6%; 24-h stimulation group: 57.2 ± 9.11% relative to the intact side of TH-positive fibers in the striatum, Figure 6; control group: 25.9 ± 4.99%; 8-h stimulation group: 49.2 ± 9.24%; 24-h stimulation group: 57.9 ± 10.6% relative to the intact side of TH-positive neurons in the SNc, Figure 7). The 24-h stimulation group displayed significant preservation of TH-positive fibers in the striatum. Additionally, the 24-h stimulation group demonstrated more preserved TH-positive neurons in the SNc than the 8-h stimulation group.

#### Iba1 Staining

**FIGURE 8:**
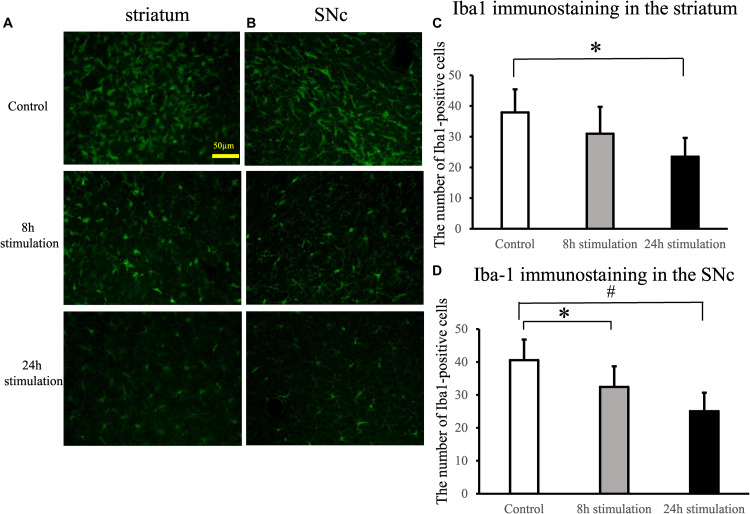
Spinal cord stimulation and Iba1 staining in the striatum and SNc. **(A,B)** Iba1 staining in the striatum **(A)** and the SNc **(B)** of the lesion side (40×). **(C,D)** The number of Iba1-positive cells in the lesioned striatum **(C)** significantly decreased in the 24-h stimulation group compared to the control group (^∗^*p* < 0.05). Similarly, the number of Iba1-positive cells in the lesioned SNc **(D)** significantly decreased in the 24-h stimulation group compared to the control group (^#^*p* < 0.01, ^∗^*p* < 0.05).

The number of Iba1-positive cells in the striatum and the SNc of rats in the 24-h stimulation group decreased significantly compared to the control group. In the 8-h stimulation group, the number of Iba1-positive cells tended to decrease in the striatum, and was significantly decreased in the SNc (control group: 37.9 ± 7.55; 8-h stimulation group: 31 ± 8.73; 24-h stimulation group: 23.5 ± 6.13 cells/field of view in the striatum; control group: 40.6 ± 6.26; 8-h stimulation group 32.4 ± 6.30; 24-h stimulation group 25.1 ± 5.62 cells/field of view in the SNc; Figure 8).

#### Laminin Staining

**FIGURE 9:**
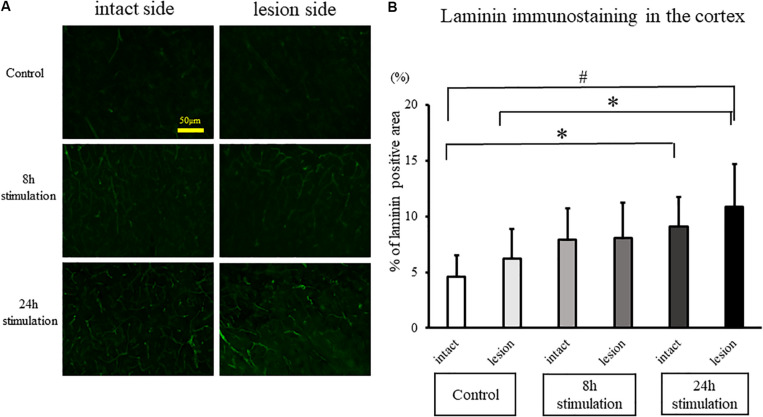
Spinal cord stimulation and laminin staining in the cerebral cortex. **(A)** Laminin-positive areas in the cerebral cortex of rats in the stimulation groups were augmented compared to those in the control group (40×). **(B)** Laminin-positive cells in the cerebral cortex in the 24-h stimulation group were augmented compared to those in the control group (^#^*p* < 0.01, ^∗^*p* < 0.05).

The laminin-positive area in the lesioned cortex significantly increased in the 8- and 24-h stimulation groups compared to the control group of the intact and lesion side (control group intact side: 4.59 ± 1.89%; control group lesion side: 6.23 ± 2.63%; 8-h stimulation group intact side: 7.90 ± 2.82%; 8-h stimulation group lesion side: 8.04 ± 3.19%; 24-h stimulation group intact side: 9.12 ± 2.58%; 24-h stimulation group lesion side: 10.8 ± 3.90%; Figure 9). Laminin-positive area in the striatum and the SNc were also explored, but there were no differences among all the groups (data not shown).

## Discussion

The present study demonstrated that a small mobile device efficiently delivered continuous SCS and exerted neuroprotective effects behaviorally and immunohistochemically on PD rats in a time-dependent manner. While both SCS-treated groups generally improved their performance in both contralateral bias and methamphetamine rotations, and displayed an increase in laminin-labeled cerebral blood vessels, The 24-h stimulation group conferred better therapeutic effects than the 8-h stimulation group, in that the longer continuous SCS regimen significantly reduced microglial cells both in the lesioned striatum and SNc compared to rats in the control group (Supplementary Figure S1).

### Small Mobile Device for Continuous SCS

Until now, conventional SCS machines allow limited control of stimulation parameter and highly restrict the movements of animals. Current SCS machines consist of a large electrical stimulator and an electrode implanted in the animals with wire connections (Maesawa et al., 2004; Spieles-Engemann et al., 2010, 2011; Santana et al., 2014; Sato et al., 2014; Shinko et al., 2014; Brys et al., 2017). Long-term adhesion of the wire to the skin results in erosion or infection of the animals. Moreover, the routine use of general anesthesia when delivering fSCS (Shinko et al., 2014) restricts free movement of the animals. Additionally, the invasive nature of current SCS procedure likely alters experimental outcomes. Because of the large size of stimulator, hard-wired connections between stimulator and electrodes, use of anesthesia, and invasive procedure, the duration and timing of electrical stimulation remain limited with conventional SCS.

A small mobile electrical stimulator may circumvent the technical limitations of current SCS machines. Indeed, such mobile device shows efficacy as a DBS apparatus for PD animals (Badstübner et al., 2012; Badstuebner et al., 2017). In this study, we developed a small mobile device for continuous SCS. This system achieved minimal invasiveness, free movement with a wireless system, easily accessible adjustment of stimulation conditions, and robust and stable stimulation for at least 2 weeks in PD animals. Notably, Bluetooth signaling efficiently controlled stimulation parameters. The present study thus extended the utility of small mobile device originally employed in DBS to SCS, the latter being less invasive with the electrode epidurally implanted as opposed to the former that targets the deep regions of the brain (e.g., thalamus, subthalamic nucleus, and globus pallidus). We envision that a closed-loop stimulation device harboring a stimulation/receiving function will allow SCS to respond in real time and in a graded manner based on the individual’s disease state. Such mobile SCS device will likely become available in the near future in view of technological developments in downsizing and wireless communication.

### Prolonged SCS Improves Therapeutic Outcomes in PD Animals

Although neuroprotective effects of SCS have been documented in PD animals, the optimal electrical stimulation conditions remain unclear. Effective electrical stimulation parameters in PD rats vary in pulse width (400–1,000 μs), frequency (300–333 Hz), stimulation duration (30 min at 2 times/week for 4.5 weeks – 30 min at once a week for 5 weeks) (Yadav et al., 2014; Brys et al., 2017). Previously we showed that the optimal conditions of “short burst” of SCS were as follows: pulse width, 100 μs; frequency, 2, 50, and 100 Hz; stimulation duration, 1 h for 16 consecutive days (Shinko et al., 2014). In the present study, we now tested the “continuous” SCS approach. Here, we confirmed that 50 Hz was the optimal frequency. To simulate the clinical settings and to reveal the time-dependency of SCS, we set stimulation duration at 8 and 24 h. Whereas behavioral amelioration, preservation of nigral TH-positive neurons, and level of angiogenesis did not differ between the 8- and 24-h stimulation groups, the longer SCS preserved more striatal TH-positive fibers and exerted better anti-inflammatory effects than the shorter SCS treatment. The dampened microglial cell activation produced by longer SCS treatment suggests that a progressive detrimental neuroinflammation may accompany PD requiring prolonged anti-inflammatory treatment to effectively sequester such cell death pathway.

### Anti-inflammatory Effects of SCS

Parkinson’s disease neurodegeneration manifests in part as a chronic neuroinflammation characterized by activated microglial cells in the striatum and SNc (Hirsch et al., 2012). Electrical stimulation may modulate neuroinflammation in that-DBS treatment in normal SD rats reduces the number of activated microglia around the electrode (Vedam-Mai et al., 2016). In tandem, SCS treatment also confers such anti-inflammation in an animal model of spinal cord ischemic reperfusion injury by reducing microglial activation through downregulation of the ERK1/2 pathway (Dong et al., 2018), a signaling pathway supported by pain studies (Morioka et al., 2013; Jiang et al., 2016; Liu et al., 2016; Huang et al., 2019; Zhong et al., 2019). In our study, SCS after intrastriatal 6-OHDA administration in the 24-h stimulation group decreased the number of microglia cells likely by exerting anti-inflammatory effects through the signaling pathways originating from the dorsal column-medial lemniscus then propagating to the SNc and striatum. Probing this anti-inflammatory signaling mechanism warrant electrophysiological experiments.

### Enhanced Angiogenesis by SCS

Low-frequency cervical SCS increases cerebral blood flow (Isono et al., 1995; Zhong et al., 2004; Yang et al., 2008), which persists up to at least 15 min after discontinuation of SCS (Isono et al., 1995). However, there has been no report about the relationship between the vasculostructural changes of cerebral blood vessels and SCS. In the present study, SCS increased the laminin-positive areas in the cerebral cortex of the lesion side compared to the control group. These results resemble the observation that intrastriatal transplantation of encapsulated VEGF-secreting cells in PD rats enhances angiogenesis (Yasuhara et al., 2004). Moreover, these findings parallel the upregulation of VEGF in the lesioned striatum of PD rats that received intermittent SCS (1 h/day for 7 consecutive days) (Shinko et al., 2014). That SCS modulates specific vasculature-associated growth factors suggests a crosstalk between electrical stimulation and growth factor secretion (Bagetta et al., 2011; Escamilla-Sevilla et al., 2011; Seifried et al., 2013; Maioli et al., 2015; Muñoz et al., 2016), which may mediate the observed increase in laminin-positive vascular area in the cerebral cortex of SCS-treated PD rats.

### Clinical Application of SCS for PD in the Future

Neuroinflammation in PD pathogenesis may involve multi-pronged neurodegenerative processes, such as inflammation and downregulation of neurotrophic factors (Yasuhara et al., 2004; Shinko et al., 2014; Chen et al., 2018; Kim et al., 2018; Troncoso-Escudero et al., 2018). This neurodegeneration plagued with aberrant inflammation and dampened neurotrophic factor levels manifests as a key secondary cell death pathway in other neurological disorders, such as stroke, traumatic brain injury, Huntington’s disease, and peripheral nerve injury (Borlongan et al., 2000; Xia et al., 2004; Emerich et al., 2006; Shojo et al., 2010; Rodrigues et al., 2012), which equally poses as a potent therapeutic target. Probing the potential of SCS to abrogate these cell death pathways may provide novel insights into the mechanism of electrical stimulation and further optimize its therapeutic outcomes.

Deep brain stimulation stands as an effective treatment for motor symptoms in advanced PD patients. SCS offers a less invasive approach compared to DBS in that the procedure spares the brain from surgical manipulations. Such minimally invasive SCS may be equally effective as DBS in reducing the hallmark PD motor deficits. Indeed, SCS alleviates motor deficits in PD marmosets (Santana et al., 2014). However, a case report shows that SCS fails to relieve akinesia or restore locomotion in two PD patients (Thevathasan et al., 2010). Optimization of SCS, including the use of continuous stimulation produced by a small mobile stimulator, may improve the clinical benefits of this minimally invasive electrical stimulation.

### Study Limitations

In this study, we used PD model of rats induced by 6-OHDA. The main advantages of this model include the ease of creating the lesion that produces loss of dopaminergic fibers in the striatum and of dopaminergic neurons in the substantia nigra. One of the disadvantages of this model is that it does not resemble the natural pathology of PD, which is slow progression of the degeneration of nigrostriatal dopaminergic neurons with degradation of α-synuclein. Therapeutic potentials of the SCS should be explored with other PD models of neurodegeneration and α-synucleinopathy reminiscent of the clinical scenario.

The aim of this study was to explore the neuroprotective effects of the SCS with duration of treatment as a factor. Here, treatment was started immediately after 6-OHDA lesion induction, which may not be applicable in the clinical setting since PD symptoms do no manifest when at least 80% of the dopaminergic neurons have already been depleted. Testing SCS in a late-stage PD model is warranted. Another limitation is that elucidating the therapeutic mechanism of SCS will require additional studies. In our study, the neuroprotective effects with angiogenic potentials were shown, but whether the neuroprotective effects of SCS during the pre-symptomatic phase is sustained during the symptomatic stage warrants further examination. In the future, behavioral changes over time after discontinuation of the SCS may reveal long-lasting effects of SCS, as well as its mechanism of actions, on PD symptoms.

## Conclusion

We demonstrated that a small mobile stimulator afforded continuous SCS and exerted neuroprotective effects in PD rats in a time-dependent manner. SCS attenuated behavioral and histological deficits associated with 6-OHDA-induced PD symptoms, possibly by mitigating microglial activation while enhancing angiogenesis. The newly developed device for continuous SCS serves as a useful tool for basic research in our understanding of interplay across electrical stimulation, neurodegeneration, and neural repair, but also advances its utility as a therapeutic modality for PD.

## Data Availability Statement

The raw data supporting the conclusions of this article will be made available by the authors, without undue reservation, to any qualified researcher.

## Ethics Statement

The animal study was reviewed and approved by Institutional Animal Care and Use Committee of Okayama University Graduate School of Medicine (Protocol# OKU-2018807).

## Author Contributions

KeK and TS contributed conception and design of the study. KeK, TY, YO, KH, IK, MO, SY, SK, YT, and MU performed the experiments. KeK and JM collected the data. KyK and NT performed the statistical analysis. KeK wrote the first draft of the manuscript. KyK, TS, TY, and J-YL wrote sections of the manuscript. CB performed the critical editing. ID supervised the study. All authors contributed to manuscript revision, read and approved the submitted version.

## Conflict of Interest

The authors declare that the research was conducted in the absence of any commercial or financial relationships that could be construed as a potential conflict of interest.

## Supplementary Material

FIGURE S1 The graphic abstract showing therapeutic effects of SCS against 6-OHDA-induced PD model of rats through angiogenesis and anti-inflammation.

VIDEO S1 The video showing twitching rats with SCS. The intensities corresponded to the 80% of motor threshold were used for each rat.

## Abbreviations

BDNF: brain-derived neurotrophic factor
DA: dopamine
DBS: deep brain stimulation
Iba1: ionized calcium-binding adaptor molecule 1
PD: Parkinson’s disease
SCS: spinal cord stimulation
SNc: substantia nigra pars compacta
TH: tyrosine hydroxylase
VEGF: vascular endothelial growth factor
6-OHDA: 6-hydroxydopamine.
