# Supplementary figures and images for: Long-Term Continuous Cervical Spinal Cord Stimulation Exerts Neuroprotective Effects in Experimental Parkinson’s Disease
Source: Front Aging Neurosci. 2020 Jun 16;12:164. doi: 10.3389/fnagi.2020.00164 (PMC7309445; doi:10.3389/fnagi.2020.00164)

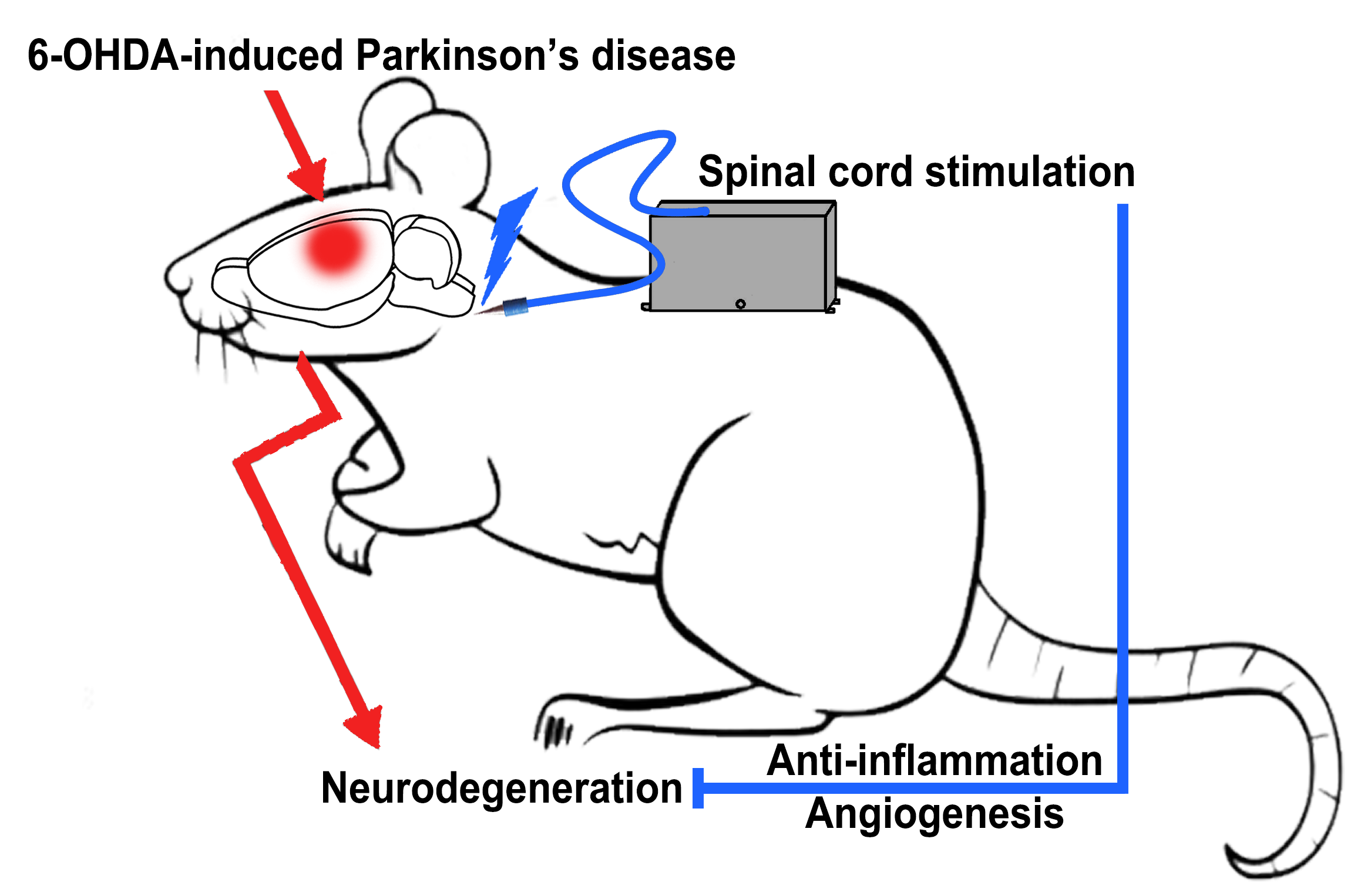

Supplement: FIGURE S1 — The graphic abstract showing therapeutic effects of SCS against 6-OHDA-induced PD model of rats through angiogenesis and anti-inflammation. [file Image_1.TIF]
